# Conversion therapy for unresectable hepatocellular carcinoma after lenvatinib: Three case reports

**DOI:** 10.1097/MD.0000000000022782

**Published:** 2020-10-16

**Authors:** Tetsu Tomonari, Yasushi Sato, Hironori Tanaka, Takahiro Tanaka, Tatsuya Taniguchi, Masahiro Sogabe, Koichi Okamoto, Hiroshi Miyamoto, Naoki Muguruma, Yu Saito, Satoru Imura, Yoshimi Bando, Mitsuo Shimada, Tetsuji Takayama

**Affiliations:** a Department of Gastroenterology and Oncology, Institute of Biomedical Sciences, Tokushima University Graduate School; b Department of Digestive and Transplant Surgery, Tokushima University; c Molecular and Environmental Pathology, University of Tokushima Graduate School, Tokushima, Japan.

**Keywords:** ablation, conversion therapy, hepatic functional reserve, hepatocellular carcinoma, lenvatinib, resection

## Abstract

**Introduction:**

Lenvatinib (LEN) is a novel potent multi-tyrosine kinase inhibitor, approved as first-line treatment for unresectable hepatocellular carcinoma (HCC). Considering its high objective response rate, LEN therapy could be expected to achieve downstaging of tumors and lead to conversion therapy with hepatectomy or ablation. However, the feasibility of conversion therapy after LEN treatment in unresectable HCC remains largely unknown.

**Patient concerns:**

Here, we reported 3 cases of unresectable HCC: case 1, a 69-year-old man diagnosed with ruptured HCC; case 2, a 72-year-old woman with nonalcoholic steatohepatitis-based HCC; and case 3, a 73-year-old man with a history of alcoholic cirrhosis-based HCC.

**Diagnosis:**

In all cases, cirrhosis was classified as Child-Pugh 5 and modified albumin-bilirubin grade 1 or 2a. HCC was diagnosed as Barcelona Clinic Liver Cancer (BCLC) stage B.

**Interventions:**

In all cases, LEN was initiated after conventional-transcatheter arterial embolization enforcement, while maintaining liver function.

**Outcomes:**

In all cases, the main tumor size decreased after 6 months of LEN treatment and no satellite nodes were detected, indicating downstaging of HCC to BCLC stage A. Subsequently, conversion hepatectomy or ablation was performed. After successful conversion therapy, the general condition of the patients was good, without tumor recurrence during the observation period (median 10 months).

**Lessons:**

This study demonstrated that LEN enables downstaging of HCC and thus represents a bridge to successful surgery or ablation therapy. In particular, LEN treatment may facilitate the possibility for conversion therapy of initially unresectable HCC, while maintaining the hepatic functional reserve.

## 1 Introduction

Hepatocellular carcinoma (HCC) is the fifth most commonly diagnosed malignancy and the second leading cause of cancer-related deaths worldwide.^[1]^ A recent phase III trial, REFLECT, indicated that lenvatinib (LEN) was non-inferior to sorafenib (SOR) as a first-line treatment for unresectable HCC.^[2]^ Based on these results, LEN was approved in Japan and other countries, as the first-line systemic treatment for patients with unresectable advanced HCC. LEN has shown a remarkably high response rate in HCC patients compared to other known molecular targeting agents (MTAs).^[2]^

It has been recently reported that conversion from unresectable to resectable metastatic gastrointestinal cancer through advances in systemic chemotherapy, termed as “conversion therapy”, could improve the prognosis of patients.^[3,4]^ Similarly, in patients with unresectable HCC, if an incurable disease apparently disappears or is well-controlled during primary treatment, surgery to excise any macroscopically remaining disease, with curative intent, could be possible. However, the feasibility and efficacy of conversion therapy in HCC remain unknown.

In this report, 3 cases of patients with unresectable HCC who successfully achieved conversion therapy are described. These patients were treated with LEN, and eventually had resection or ablation after showing good clinical and radiological response.

## 2 Case reports

Table 1 provides the summary of clinical characteristics and courses of 3 patients with unresectable HCC who underwent conversion therapy after LEN treatment. Written informed consent was obtained from the 3 patients for publication of this case report.

**Table 1:**
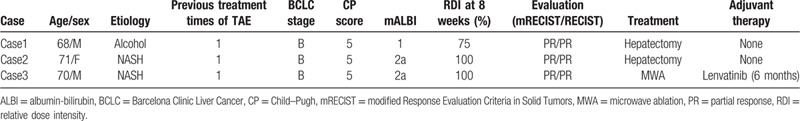
Clinical characteristics and courses of patients with unresectable HCC who achieved conversion therapy.

### 2.1 Case 1

**Figure 1:**
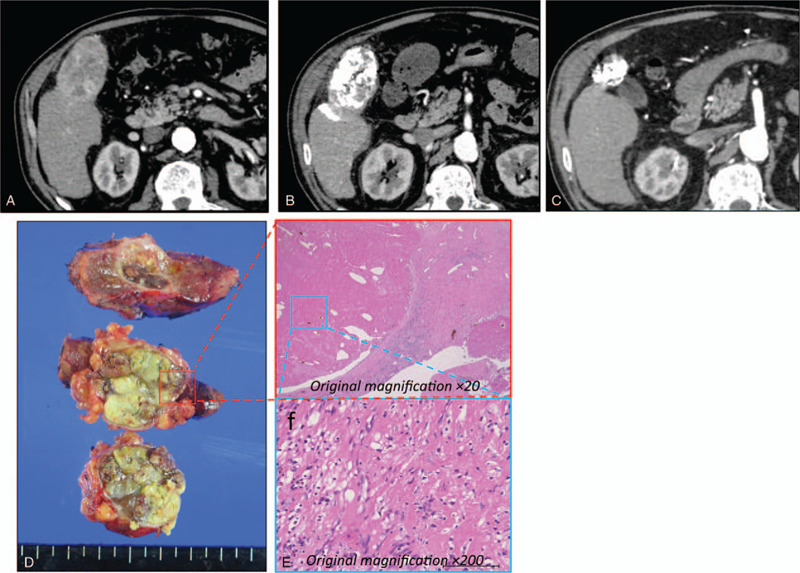
Clinical images of ruptured hepatocellular carcinoma in a 69-year-old man (case 1). (a) Dynamic computerized tomography (CT) findings at initial diagnosis. CT images showed a large hypervascular liver tumor in segment 6, measuring 7.3 cm. (b) CT findings after conventional-transcatheter arterial embolization treatment. (c) CT findings after 6 months of LEN treatment showed that the tumor burden had decreased. (d) Image of the resected specimen obtained after laparoscopic hepatectomy. (e, f) Histological findings from the main tumor (hematoxylin and eosin stain). There were no tumor cells on resection margins. Pathological diagnosis revealed a necrotic nodule.

A 69-year-old man, with a history of alcoholic cirrhosis, presented with abdominal pain. The cirrhosis was classified as Child-Pugh 5 and modified ALBI (mALBI) grade 2A. Computed tomography (CT) showed a large hypervascular liver tumor, measuring 7.3 cm. Many satellite nodules had disseminated in all the liver segments (Fig. 1a). Based on the presence of typical arterial uptake, with portal wash-out on dynamic CT and elevated α-fetoprotein (AFP) level (1637 ng/ml; normal <10 ng/ml), HCC in BCLC stage B was diagnosed.

Further, since the tumor had extrahepatic growth accompanied by abdominal pain and progression of anemia and ascites was seen, this case was diagnosed as ruptured HCC. Therefore, an emergency conventional-transcatheter arterial embolization (cTAE) and angiography, targeting the main tumor, were performed (Fig. 1b).

However, a follow-up CT evaluation after 1 month revealed that lipiodol was washed out from more than half of the tumor, and the tumor marker level increased from 866 to 1637 mg/dl. In addition, there were concerns about the possible locoregional recurrence and peritoneal seeding. Thus, LEN was orally administrated at 12 mg/day. No severe side effects were observed, except grade 2 hypertension and palmar-plantar erythrodysesthesia at days 24 and 36, respectively. Two months after the initiation of LEN therapy, the patient had partial response (PR), according to the response evaluation criteria in solid tumors (RECIST) and the modified RECIST (mRECIST) criteria. Additionally, the AFP level markedly decreased from 1637 to 4 ng/ml and was sustained within the normal range with continued therapy. After 6 months of LEN treatment, a follow-up CT examination showed that the tumor burden had significantly decreased, along with the presence of extensive necrotic areas; thus suggesting antitumor activity of LEN, especially as an angiogenesis inhibitor (Fig. 1c). We further confirmed by CT and magnetic resonance imaging that there were no satellite nodules in the liver tissue surrounding the tumor.

Based on these results, it was concluded that the intermediate-stage HCC (BCLC stage B) was downstaged to an early stage HCC (BCLC stage A). A laparoscopic hepatectomy was performed for the main nodule. The macroscopic and microscopic histopathological examinations showed necrosis of HCC with no tumor cells observed on the resection margins, and the pathological diagnosis was necrotic nodule (Fig. 1d-f). There was no clinical evidence of recurrence at 6 months after the resection. The AFP levels remained within the normal limits.

### 2.2 Case 2

**Figure 2:**
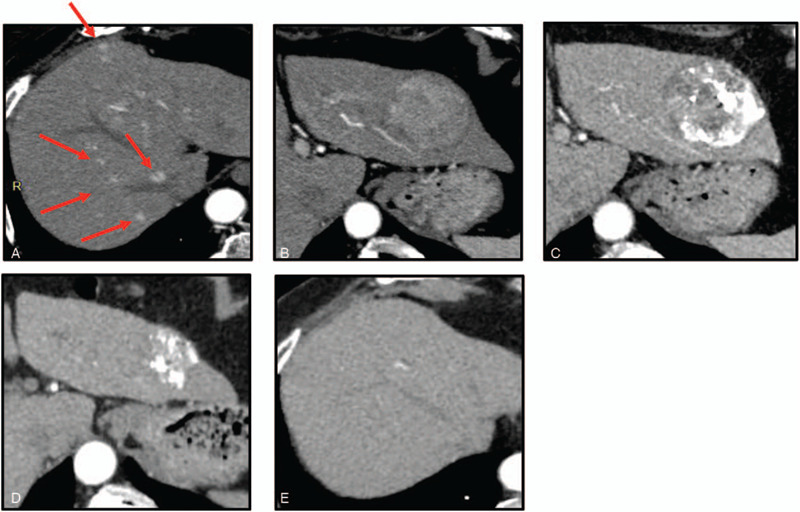
Clinical images of nonalcoholic steatohepatitis-based hepatocellular carcinoma in a 72-year-old woman with (case 2). (a, b) Dynamic computerized tomography (CT) findings at initial diagnosis showed a large hypervascular liver tumor in S3, measuring 5.8 cm and many satellite nodules, disseminated in all the liver segments. (c) CT findings after 1 month showed that lipiodol was washed out from more than half of the tumor lesion. (d, e) CT findings after 6 months. The main tumor size had decreased. Extensive necrotic areas were observed, while no satellite nodes were detected.

A 72-year-old woman had been followed-up for nonalcoholic steatohepatitis. The cirrhosis was classified as Child-Pugh 5 and mALBI grade 2A. A liver tumor was detected by periodic abdominal ultrasonography. Abdominal dynamic CT demonstrated an enhancement in the arterial phase and wash-out in the late phase. Large hypervascular liver tumor in S3, measuring 5.8 cm, was detected along with many satellite nodules that were disseminated in all the liver segments (Fig. 2a, b). Based on the presence of typical arterial uptake with portal wash-out on dynamic CT, a typical hypervascular HCC in BCLC stage B was diagnosed. We initially performed cTAE, targeting the main tumor. However, a CT examination after 1 month showed that lipiodol was washed out from more than half of the tumor lesion (Fig. 2c) and the tumor marker level increased rapidly. We then decided to switch to MTA treatment to maintain sufficient hepatic reserve function. LEN was orally administered at 8 mg/day. Hypertension (Grade 2) was observed at day 49 after the initiation of LEN therapy. After about 2 months of LEN treatment, the patient exhibited PR (according to RECIST and the mRECIST criteria). After 6 months of treatment, the main tumor size had decreased considerably, with extensive necrotic areas observed (Fig. 2d). No satellite nodes were detected (Fig. 2e).

Although no obvious staining lesion was observed, there was still a concern that viable cells might remain in the main lesion. Once the BCLC stage B HCC was downstaged to BCLC stage A, we decided to perform left hepatectomy at 6 months after the initiation of LEN therapy. LEN was discontinued for 10 days before the surgery to prevent surgical complication.

**Figure 3:**
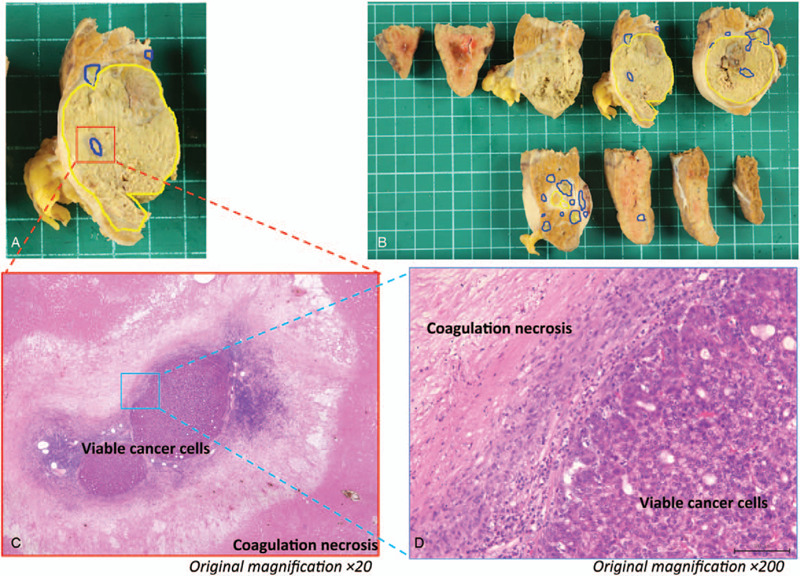
Clinical images of nonalcoholic steatohepatitis-based hepatocellular carcinoma in a 72-year-old woman with (Case 2). (a, b) The resected specimen obtained after laparoscopic hepatectomy. The sections within the blue colored lines are viable cancer cells, while the sections within the yellow colored lines are necrotic cells. (c, d) Histological findings from the main tumor (hematoxylin and eosin stain).

The pathological diagnosis was moderate type HCC (Fig. 3a-d). Finally, the patient was determined to have pathological stage I disease, and curative resection was achieved. There were no serious complications. At 3 months after hepatectomy, the patients general condition was good and no tumor recurrence was observed.

### 2.3 Case 3

**Figure 4:**
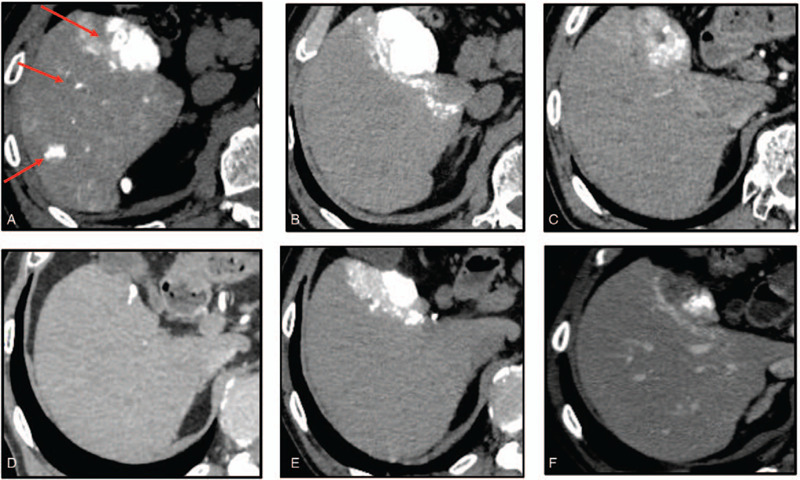
Clinical images of alcoholic cirrhosis-based hepatocellular carcinoma in a 73-year-old man (Case 3). (a, b) Dynamic computerized tomography (CT) findings at initial diagnosis showed a large hypervascular liver tumor in S5, measuring 5.2 cm, and many satellite nodules in S5/6 the liver segments. (c) CT findings after 1 month showed that lipiodol was washed out from more than half of the tumor lesion. (d) CT findings after 6 months showed that the main tumor size had decreased. (e) CT findings after conventional-transcatheter arterial embolization treatment. (f) CT findings after treated with microwave ablation.

A 73-year-old man presented with a history of alcoholic cirrhosis. The cirrhosis was classified as Child-Pugh 5 and mALBI grade 1. A dynamic CT scan showed a typical hypervascular HCC in S5, measuring 5.2 cm, and many satellite nodules located in S5/6 (Fig. 4a). Therefore, BCLC stage B was diagnosed. The patient was initially treated with cTAE, targeting the main tumor (Fig. 4b). However, follow-up CT examination after 1 month revealed that more than half of the lipiodol was washed out from the target tumor (Fig. 4c), suggesting that the tumor would eventually become refractory to cTAE treatment. Therefore, we switched the treatment to LEN monotherapy (oral administration at 12 mg/day). No severe side effects were seen, except for grade 2 hypertension. After 2 months of LEN treatment, PR was observed (according to the RECIST and mRECIST criteria). LEN therapy was continued, and the patients general condition was good. After 6 months of treatment, subsequent CT scans revealed that the target lesion size in the main tumor decreased from 5.2 cm to 2 cm and no satellite nodes were detected (Fig. 4d). Therefore, it was concluded that HCC in BCLC stage B was downstaged to BCLC stage A. LEN treatment was interrupted for 1 week and the patient underwent cTAE on the target lesion. Subsequently, microwave ablation was performed in combination with artificial ascites (Fig. 4e, f). Next day, complete cauterization of the target lesion was confirmed by CT evaluation. LEN treatment was resumed after a 2-week interruption. No recurrence was observed after 6 months of MTA treatment.

## 3 Discussion

Conversion therapy has been established as a therapeutic strategy for the treatment of patients with gastrointestinal cancer with unresectable metastases, controlled by chemotherapy and additional surgical resection. However, the feasibility and efficacy of conversion therapy in HCC have not been studied well. Considering its high objective response rate and low impact on liver function, LEN therapy could be expected to achieve downstaging of tumors and lead to conversion therapy with surgical procedure.

In this context, we defined the indication of conversion therapy in patients who could achieve downstaging of HCC from BCLC stage B to BCLC stage A after treatment with LEN. Data from 3 patients, who successfully achieved conversion therapy by surgical resection or ablation, are presented in this report, and the clinical characteristics and courses are summarized in Table 1.

Before the approval of LEN, SOR was prescribed as the first-line treatment for HCC. To date, there have been few reported cases of HCC patients who underwent conversion therapy after SOR treatment, leading to long-term survival.^[5–7]^ This may be due to the fact that SOR monotherapy can rarely downstage unresectable HCC to resectable stage, due to a low response rate of 12.4%, according to the mRECIST.^[2]^ In contrast, LEN has demonstrated a significantly higher response rate than other MTAs, which is reported to be extremely high at 40.6% by independent imaging review (according to the mRECIST).^[2]^ The high tumor shrinkage rate of LEN might have resulted in the downstaging of HCC patients. Additionally, the maintenance of better liver function in all the patients before the initiation of LEN treatment could have contributed to the downstaging in these patients. These results reiterate the observation that better hepatic reserve function is associated with the therapeutic effect of LEN.^[8–10]^ It has also been reported that the relative dose intensity (RDI) plays an important role in the therapeutic effect of LEN.^[8,11,12]^ In all of our cases, hepatic reserve function was good and the RDI, up to the first imaging evaluation, was high (Case 1: 75%, Case 2: 100%, Case 3: 100%) (Table 1). Although in BCLC stage B, cTACE/TAE is a standard treatment for unresectable HCC, Arizumi et al and Ogasawara et al reported that the switch from cTACE to SOR significantly improved the OS in patients with intermediate-stage HCC who were refractory to cTACE therapy. Therefore, they suggested that administration of SOR is recommended in patients who are unresponsive to cTACE, when appropriate.^[13,14]^ Furthermore, Kudo et al have reported that it is more effective to use LEN before cTACE in BCLC stage B cases, suggesting the importance of early initiation of LEN therapy.^[15]^ Considering these reports, we initially performed cTAE for the main lesion, but only once so as to avoid the development of resistance to TAE, and soon switched to LEN monotherapy, which resulted in high response efficiency in all the cases (Table 1).

For HCC patients, mRECIST is often used for the standard assessment of treatment efficacy; particularly in patients who receive antiangiogenic drugs.^[16]^ However, there is some concern as to whether mRECIST is appropriate to evaluate the resectability of HCC, since it does not necessarily reflect the actual tumor shrinkage and only evaluates the elimination of tumor staining. In our cases, we used both mRECIST and RECIST criteria to confirm the high response rate, which led to the decision of conversion therapy. In the resected specimens from cases 1 and 2, the tumors became necrotic even at the site where the tumor stain disappeared in mRECIST evaluation. In addition, histopathological examination showed almost no residual tumor in the resected liver tissues (Figs. 1 and 3). These results demonstrated the usefulness of mRECIST in deciding the resectability of tumors. However, further data is required to verify this result.

The efficacy and safety of SOR treatment in HCC patients with spontaneous rupture have been reported previously.^[17]^ However, there were no reports on LEN treatment in this patient population. Rupture of HCC can lead to peritoneal dissemination and recurrence. Therefore, we suggest that LEN should be available for HCC patients with spontaneous rupture, as presented in case 1.

LEN targets various receptors, including VEGF 1–3, FGF 1–4, PDGF α, RET, and KIT.^[18–20]^ Hence, LEN treatment is discontinued peri-operatively if surgical or ablation procedures are required, since the antiangiogenic effects of LEN can result in complications, such as delayed healing and bleeding. However, there is no established protocol for the appropriate timing of LEN interruption. Since the half-life of LEN is approximately 28 to 35 h,^[21]^ the duration of interruption of pre-operation or pre-ablation needs to be at least 1 week, considering the safety margin. The pre-operation or pre-ablation was 7 days in cases 1 and 3, and 64 days in case 2. The latter needed urgent pacemaker implantation for the treatment for the AV block, unrelated to the administration of LEN. As a result, no obvious complications were observed after the operation in all the cases.

It is not clear whether continuation of LEN treatment after resection of HCC would be effective, since the results of the STORM study (SOR administered to HCC patients in postoperative setting) were negative.^[22]^ Therefore, we administered adjuvant LEN treatment only to the patient who received ablation treatment (case 2), since we could not completely rule out the possibility of the presence of residual tumor. After 6 months of LEN monotherapy, treatment was discontinued once no recurrence was confirmed. In all the cases, no recurrence was seen during the observation period (median 8 months, range 6–8). However, further prospective studies may be needed to demonstrate whether adjuvant LEN is required after conversion therapy.

Since this report is limited by its short observation period and retrospective nature, our results do not necessarily demonstrate that surgical resection or ablation improve overall survival per se. Therefore, in the future, more studies need to be conducted to investigate the therapeutic efficacy of conversion therapy using LEN.

## 4 Conclusions

In conclusion, the results from this study demonstrated that LEN enables downstaging of HCC, and thus represents a bridge to successful surgery or ablation therapy. In particular, cases with Child-Pugh 5 and m-ALBI 1, 2a were more likely to be able to switch to conversion therapy. Overall, the results indicate that treatment with LEN may improve the possibility for resection of unresectable HCC, while maintaining better hepatic functional reserve. Therefore, the initial incurability of patients with unresectable HCC should be reconsidered when the patients respond well to LEN because long-term survival may be expected in these patients with conversion therapy. Nevertheless, the results from this study need to be further validated in large scale prospective studies.

## Author contributions

All authors contributed to the study conception and design. Data collection, and analysis were performed by Tetsu Tomonari, Hironori Tanaka, Takahiro Tanaka, Tatsuya Taniguchi, Masahiro Sogabe, Koichi Okamoto, Hiroshi Miyamoto, Naoki Muguruma, Yu Saito, Satoru Imura, Yoshimi Bando, and Mitsuo Shimada. The first draft of the manuscript was written by Tetsu Tomonari, Yasushi Sato, and Tetsuji Takayama, and all authors commented on previous versions of the manuscript. All authors read and approved the final manuscript.
